# LncSHRG promotes hepatocellular carcinoma progression by activating *HES6*

**DOI:** 10.18632/oncotarget.19906

**Published:** 2017-08-03

**Authors:** Ying-Chen Xu, Chao-Jie Liang, Dong-Xin Zhang, Guan-Qun Li, Xia Gao, Jian-Zhu Fu, Feng Xia, Jia-Jun Ji, Li-Jun Zhang, Guang-Ming Li, Ji-Xiang Wu

**Affiliations:** ^1^ Department of General Surgery, Beijing Tongren Hospital, Capital Medical University, Beijing 100730, China

**Keywords:** HCC, lncSHRG, SATB1, HES6, proliferation

## Abstract

Hepatocellular carcinoma, one of the most common cancers, leads to mass mortality worldwide currently. However, the underlying mechanism of its oncogenesis remains to be elucidated. Here we identified that a long noncoding RNA, *lncSHRG*, was greatly upregulated in human hepatocellular carcinoma samples. We found that lncSHRG was essential for liver cancer cell proliferation and tumor propagation in mice. In mechanism, *lncSHRG* recruits SATB1 to bind to *HES6* promoter and initiates *HES6* expression. HES6, which is highly expressed in hepatocellular carcinoma, promotes tumor cell proliferation. High expression level of HES6 is positively correlated with clinical severity and poor prognosis of people with hepatocellular carcinoma. Altogether, our research provides a new insight on the mechanism of hepatocellular carcinoma progression.

## INTRODUCTION

As one of the most common tumors currently, hepatocellular carcinoma (HCC) is of poor prognosis and high recurrence and leads to mass mortality worldwide [1]. Many factors can give rise to HCC, such as hepatitis B virus infection and biliary disease [2, 3]. However, the underlying molecular mechanism of hepatocarcinogenesis is largely unknown. Hepatic resection and chemotherapy are the main approach in HCC therapy, but the tumor recurrence rate is especially high [4]. Up to now, there is no well-established way to heal patients with HCC [5]. Therefore, the molecular mechanism in hepatocarcinogenesis and progression urgently needs to be explored.

Long non-coding RNAs (lncRNAs) are longer than 200nt and have no coding ability [6]. Most reported lncRNAs are expressed in specific cell types. More and more research has shown that lncRNAs act as regulators for gene expression and participate in large kinds of important biological processes [6], such as tumor formation, embryonic development and immune cell lineage commitment [7–9]. Expression dysregulation of lncRNAs can contribute to human cancers, including gastric cancer [10], breast cancer [11], hepatocellular carcinoma [12] and so on. However, how lncRNAs regulate liver cancer remains elusive.

Hes family bHLH transcription factor 6 (HES6) is regarded as a target gene in Notch signaling pathway [13]. HES6 promotes cell proliferation and migration in some tumor such as glioma [14] and prostate cancer [15]. Nevertheless, the role of HES6 in HCC remains to be elaborated and the mechanism that controls its expression in a Notch signaling dependent or independent fashion has not been demonstrated.

In this study, we investigated the function of lncSHRG in liver cancer. Interestingly, our results showed that lncSHRG promotes cancer cell proliferation and tumor propagation in mice. LncSHRG recruits SATB1 to *HES6* promoter and initiated its expression. The expression level of HES6 is positively correlated with clinical severity and poor prognosis of HCC. Our study provides a new perspective on the mechanism of hepatocarcinogenesis and finds a new potential target for HCC treatment.

## RESULTS

### LncSHRG is highly expressed in HCC

**Figure 1:**
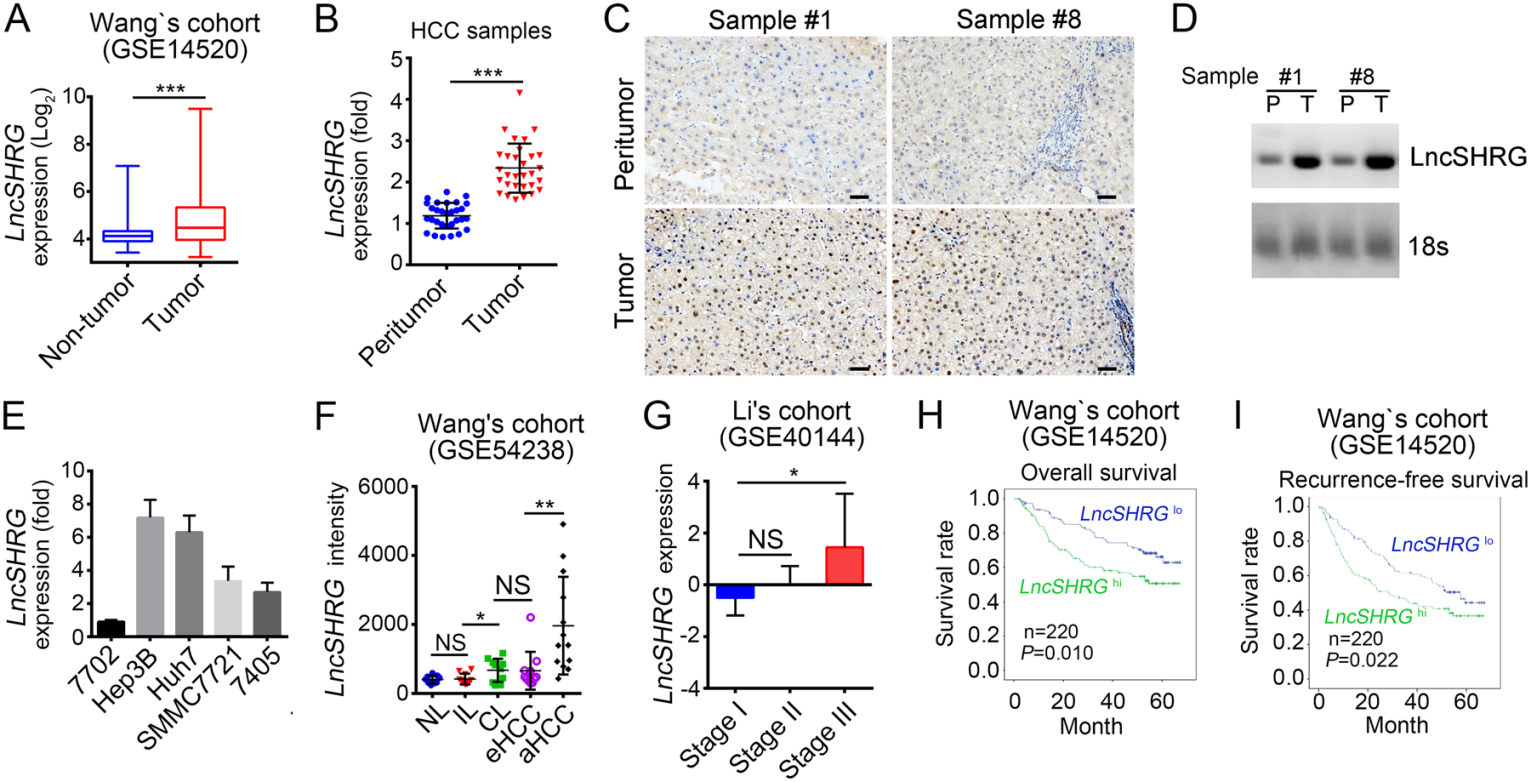
*LncSHRG* is highly expressed in HCC **(A)** Analysis of *lncSHRG* expression in peritumor and tumor tissues according to the microarray data in Wang’s cohort (GSE14520). **(B)** 30 pairs of peritumor and tumor HCC samples were collected. Then *LncSHRG* expression levels were analyzed in these sample tissues by RT-qPCR. Fold changes were normalized to endogenous *ACTB*. (**C)** LncSHRG expression in peritumor and tumor tissues of sample #1 and #8 was checked by RNA hybridization *in situ* with biotin-labeled lncSHRG probes. Scale bars, 100μm. **(D)** Total RNAs were extracted from peritumor and HCC samples. LncSHRG and 18S rRNA (loading control) was examined by Northern blot. P: peritumor; T: tumor. **(E)** Total RNAs were extracted from indicative human HCC cell lines and lncSHRG expression was checked by RT-qPCR. Fold changes were normalized to endogenous *ACTB*. **(F)** Higher expression of lncSHRG in HCC samples. LncSHRG expression was analyzed with R language and Bioconductor according to the microarray data in Wang’s cohort (GSE54238). NL: normal livers; IL: chronic inflammatory livers; CL: cirrhotic livers; eHCC: early HCC; aHCC: advanced HCC. **(G-I)** LncSHRG expression levels were positively correlated with clinical stages and poor prognosis by expression analysis (G) and Kaplan–Meier survival analysis (H and I) according to the microarray data in Li’s cohort (GSE40144) and Wang’s cohort (GSE14520). *p<0.05, **p<0.01 and ***p<0.001 by two-tailed Student’s *t* test. All data presented are shown as means ± SD collected from three independent experiments.

To understand how lncRNAs regulate the occurrence and progression of hepatocellular carcinoma, we analyzed several online-available data sets on human HCC samples in the NCBI database. Based on the sample size, type and clinical information, we finally chose 3 sets of data including GSE14520, GSE54238 and GSE40144 [16–18]. According to GSE54238 and GSE40144, we screened out an uncharacterized and 613 nucleotides long lncRNA that is highly expressed in human HCC samples and whose official gene symbol is LOC654342 located in chromosome 2. We called it *lncSHRG* for abbreviation of SATB1 and HES6 related gene. We then validated its high expression in HCC samples according to GSE14520 (Figure 1A). To investigate the role of *lncSHRG*, we firstly collected 30 pairs of peritumor and tumor samples containing detailed information about disease characteristics (Supplementary Table 1). We analyzed the expression levels of *lncSHRG* and found it was expressed nearly two times higher in tumor cells than peritumor cells (Figure 1B). Based on the expression level of *lncSHRG*, we chose sample #1 and #8 for RNA hybridization *in situ* and Northern blot. We found that *lncSHRG* was expressed higher in tumor tissues (Figure 1C and 1D). Besides, *lncSHRG* was also highly expressed in hepatocellular carcinoma cell lines including Hep3B cells, 7704 cells, Huh7 cells, SMMC7721 cells and 7405 cells but not in 7702 cells (Figure 1E). Furthermore, after analysis of microarray data in Wang’s cohort (GSE54238) and Li’s cohort (GSE40144), we found that *lncSHRG* is highly expressed in HCC tissues, especially in advanced and stage III HCC samples (Figure 1F and 1G). Importantly, according to the microarray data in Wang’s cohort (GSE14520), HCC patients with higher *lncSHRG* expression levels had lower overall and recurrence-free survival rates, and vice versa (Figure 1H and 1I). Altogether, *lncSHRG* was highly expressed in HCC samples and positively correlated with clinical severity and poor prognosis.

### LncSHRG is required for liver cancer cell proliferation and migration

**Figure 2:**
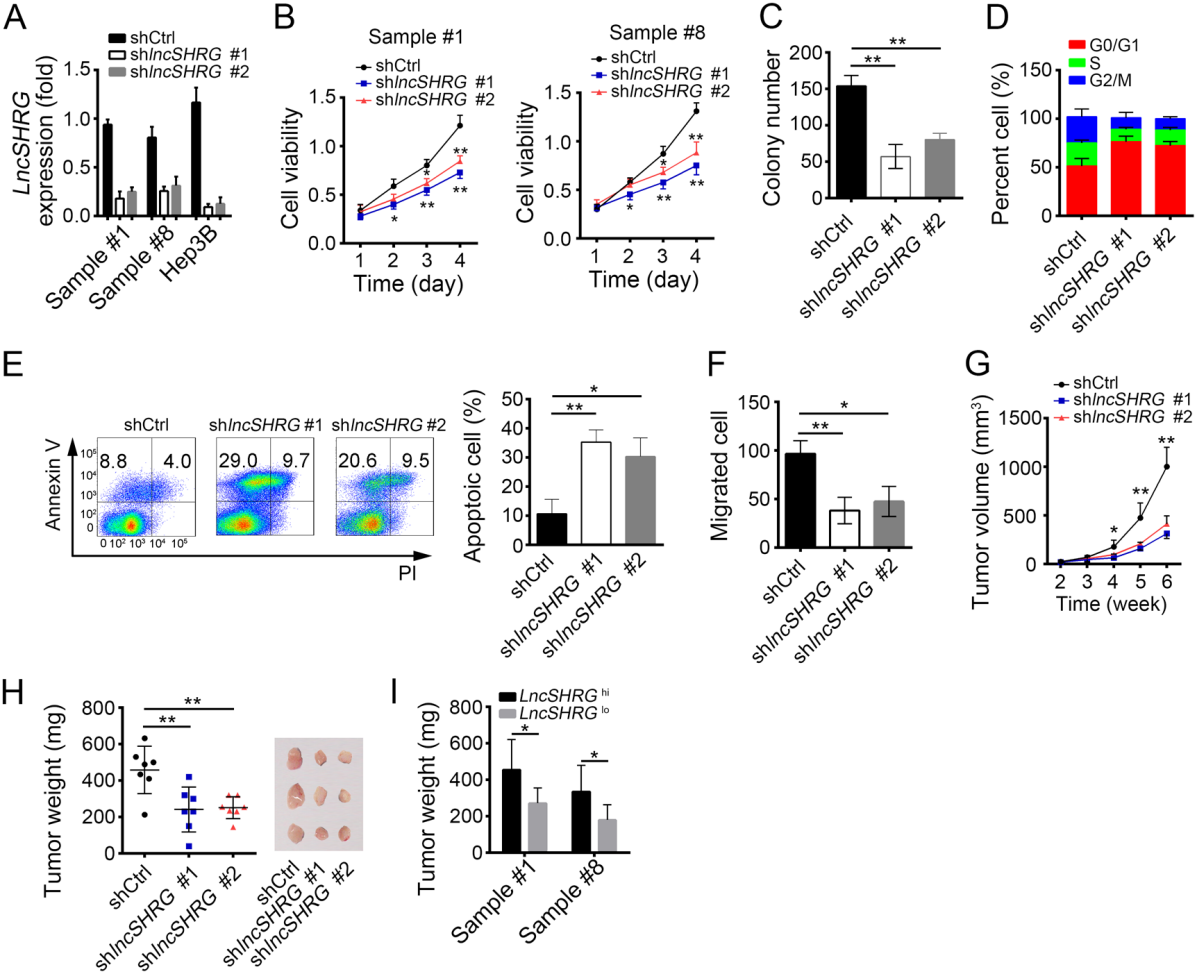
LncSHRG is required for liver cancer cell proliferation and migration **(A)** LncSHRG was knocked down in HCC sample #1 and #8, and Hep3B cells using two independent siRNA sequences by infection of lentivirus containing pSICOR-GFP-shlncSHRG. GFP-positive cells were isolated by FACS and cultured. Knockdown efficiency of lncSHRG was checked by real time qPCR. Cells infected with lentivirus containing pSICOR-GFP-scramble were chosen as control. **(B** and **C)** Effect of lncSHRG knockdown on proliferation of HCC samples was measured by MTT assays and colony formation assays. **(D)** Cell-cycle distribution of HCC sample #1 cells was analyzed after lncSHRG knockdown by FACS. **(E)** Cell apoptosis was checked in lncSHRG-depleted HCC sample #1 cells by FACS. Cells were stained with Annexin V/PI. **(F)** Effect of lncSHRG on Hep3B cell migration was analyzed by transwell assays. **(G** and **H)** The volumes and weights of tumors were measured at indicated time points. 2×10^6^ lncSHRG-depleted or control Hep3B cells were injected into nude mice. The weights of tumors were measured on week 5 after injection. **(I)** LncSHRG was important for HCC propagation. Sample cells were classed into 2 groups based on the expression level of lncSHRG. Then 2×10^6^ lncSHRG highly expressed or lowly expressed sample cells were injected into nude mice and tumor weights were measured on week 5. N=6 for each group. *p<0.05 and **p<0.01 by two-tailed Student’s t test. All data presented are shown as means ± SD collected from three independent experiments.

To define the function of *lncSHRG*, we knocked it down in HCC samples and Hep3B cells. Then we confirmed the efficiency of lncSHRG knockdown by qPCR (Figure 2A). Using MTT assays, we found that lncSHRG-depletion seriously inhibited HCC cell proliferation (Figure 2B). Besides, lncSHRG-silenced cells showed lower colony formation ability (Figure 2C). Above data indicated that lncSHRG promotes cell proliferation, which was further confirmed by the observation that lncSHRG-silenced cells were arrested in G0/G1 phase more (Figure 2D). However, lncSHRG knockdown resulted in increased cell apoptosis (Figure 2E). Then we explored the effect of lncSHRG on the ability of cell migration, and found that lncSHRG-silenced cells showed attenuated potential to migrate (Figure 2F). To investigate the function of lncSHRG *in vivo*, we injected 2×10^6^ lncSHRG-silenced Hep3B cells or control cells into BALB/c nude mice. And tumor volumes and weights were measured at indicative time points (Figure 2G and 2H). The results demonstrated that lncSHRG was required for tumor growth. What’s more, *lncSHRG* lowly expressed sample cells also showed slower growth rate *in vivo* than that with high expression level (Figure 2I). In summary, lncSHRG is essential for tumor cell proliferation.

### LncSHRG positively regulates liver cancer cell through HES6

**Figure 3:**
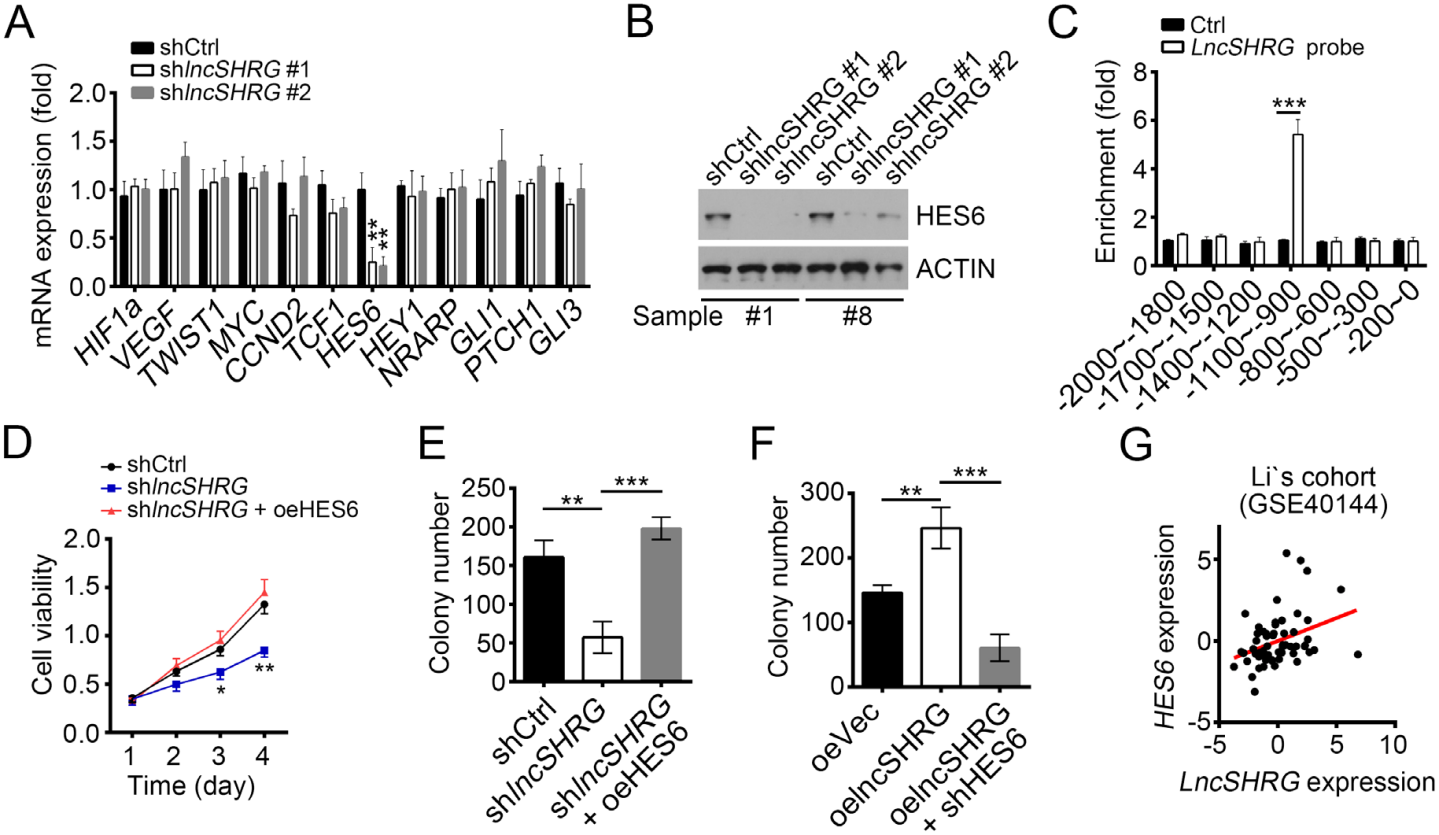
LncSHRG positively regulates liver cancer cell through HES6 **(A)** LncSHRG knockdown impaired *HES6* expression. Indicative gene expression levels in shCtrl or shlncSHRG HCC samples were analyzed by RT-qPCR. **(B)** HES6 protein levels were downregulated in HCC sample #1 and #8 cells after lncSHRG depletion. **(C)** LncSHRG bound to *HES6* promoter by CHIRP assay. Biotin-labeled lncSHRG probes were designed online and purchased from Invitrogen. HCC sample cells were lysed and genomes were sonicated into 300–500 bp DNA fragments. Then lysates were incubated with lncSHRG probes for enrichment of lncSHRG and DNA fragments. **(D** and **E)** Cell proliferation ability was analyzed by MTT assays and colony formation assay.GFP-positive sample #1 cells infected with pSICOR-GFP-shlncSHRG or pSICOR-GFP-scramble were chosen for assays. For HES6 overexpression, sample cells containing pSICOR-GFP-shlncSHRG were transfected with pCDNA3-HES6 plasmid using Lipofectamine 2000 (Invitrogen, USA). **(F)** HES6 knockdown inhibited colony formation. **(G)** *LncSHRG* expression was positively correlated with that of *HES6* in HCC samples. *p<0.05, **p<0.01 and ***p<0.001 by two-tailed Student’s t test. All data presented are shown as means ± SD collected from three independent experiments.

It is generally acknowledged that NF-κB, Wnt/β-catenin, Notch and Hedgehog signalling are essential for tumor genesis and/or maintenance. To reveal the molecular mechanism of *lncSHRG* regulating liver cancer, we analyzed the effect of *lncSHRG* knockdown on these pathways. We checked the expression levels of NF-κB target genes (*HIF1A*, *VEGFA*, *TWIST1*), Wnt/β-catenin target genes (*MYC*, *CCND1*), Hedgehog target genes (*GLI1*, *PTCH1*, *GLI3*) and Notch target genes (*HES6*, *HEY1*, *NRARP*) by RT-qPCR, and found that *lncSHRG* knockdown downregulated *HES6* expression (Figure 3A), which was further verified by western blot (WB) (Figure 3B). To explore whether *lncSHRG* regulates *HES6* expression directly, we performed chromatin isolation by RNA purification (CHIRP) assays using biotin-labeled lncSHRG specific probes and found that lncSHRG deposited on *HES6* promoter (-1100∼-900) (Figure 3C). Then we wondered whether overexpressing HES6 can rescue the effect produced by *lncSHRG* knockdown. We found that HES6 overexpression in *lncSHRG*-silenced samples enhanced the capacities of cell proliferation and colony formation to the level of control (Figure 3D and 3E). On the contrary, overexpressing lncSHRG promoted colony formation, but simultaneously knocking down HES6 reversed it (Figure 3F). Finally, we analyzed the expression correlation between *lncSHRG* and *HES6*, and found that they were positively correlated (Figure 3G). Collectively, lncSHRG directly promotes *HES6* expression in HCC cells.

### LncSHRG associates with SATB1

**Figure 4:**
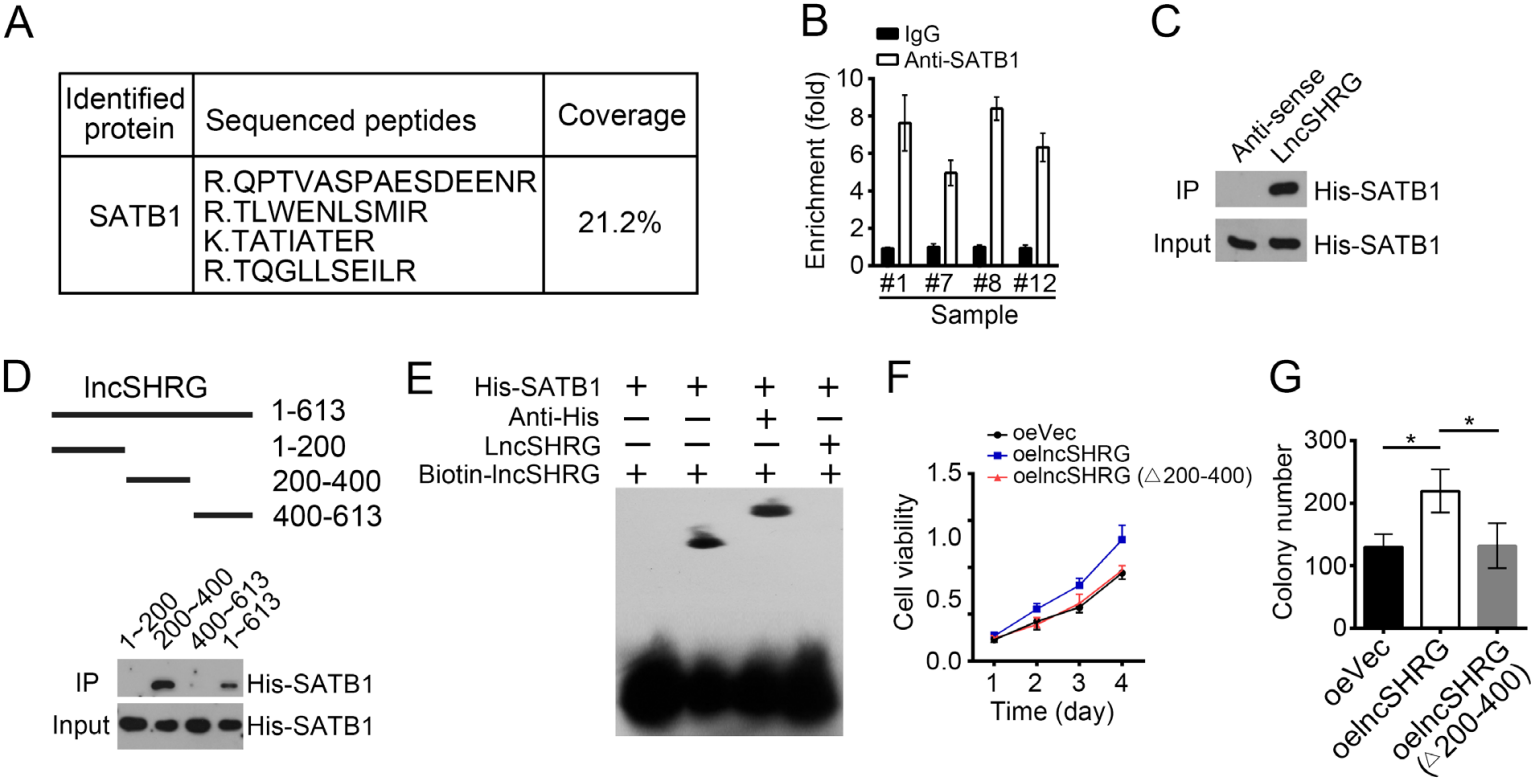
LncSHRG associates with SATB1 **(A)** Biotin-RNA pulldowns were performed using biotin-labeled lncSHRG or anti-sense control. Eluted fractions were resolved by SDS-PAGE, followed by silver staining and mass spectrometry. SATB1 was identified as a potential interactive protein of lncSHRG. **(B)** HCC sample lysates were incubated with anti-SATB1 at 4°C for 4 h, followed by an RNA immunoprecipitation assay. **(C)** Interaction of His-SATB1 with biotin-lncSHRG was checked by RNA pulldown assays. **(D)** lncSHRG (nt200∼400) was essential for the interaction with SATB1 as shown by domain mapping and RNA pulldown assays. **(E)** Biotin-labeled lncSHRG (nt200∼400) probe was incubated with His-SATB1 protein, followed by EMSA assays. **(F)** LncSHRG (nt200∼400) contributed to cell proliferation by MTT assays. Full-length lncSHRG but not truncation version (deletion of nt200∼400) promotes cell proliferation in HCC samples. **(G)** LncSHRG (nt200∼400) promoted colony formation. Full-length lncSHRG but not truncation version (deletion of nt200∼400) promotes colony formation of HCC sample cells. *p<0.05 by two-tailed Student’s t test. All data presented are shown as means ± SD collected from three independent experiments.

LncRNAs that possess flexible and modular scaffold quality can tether protein factors to regulate gene expression widely [19]. To further determine how lncSHRG promotes *HES6* expression, we prepared HCC sample cell lysates and applied to biotin-labeled lncSHRG or control (anti-sense of lncSHRG) for RNA pulldown assays. Eluted fractions were resolved by SDS-PAGE and silver staining. Then the major differential band appeared in biotin-labeled lncSHRG containing lysates were analyzed by mass spectrometry. SATB1 was identified as a potential interactive protein of lncSHRG (Figure 4A and Supplementary Figure 1A). We then confirmed this result by RNA IP and RNA pulldown assays. Endogenous lncSHRG was enriched by anti-SATB1 (Figure 4B) and Biotin-labeled lncSHRG achieved by RNA transcription *in vitro* also precipitated His-SATB1 (Figure 4C). To search the interactive region in lncSHRG with SATB1, we performed domain mapping assays. We truncated lncSHRG every other 100nt and found that neither can interact with SATB1 (Supplementary Figure 1B). Then we truncated lncSHRG every 200nt and conducted RNA pulldown assays. We found that lncSHRG (nt200∼400) was essential for its interaction with SATB1 (Figure 4D), which was further confirmed by RNA-EMSA assays (Figure 4E). Unlike lncSHRG full-length, overexpressing lncSHRG truncation version (deletion of nt200∼400) cannot promote HCC cell proliferation (Figure 4F) and colony formation (Figure 4G). In a word, lncSHRG (nt200∼400) was essential for SATB1 recruitment and its function well.

### LncSHRG is essential for SATB1 binding to *HES6* promoter

**Figure 5:**
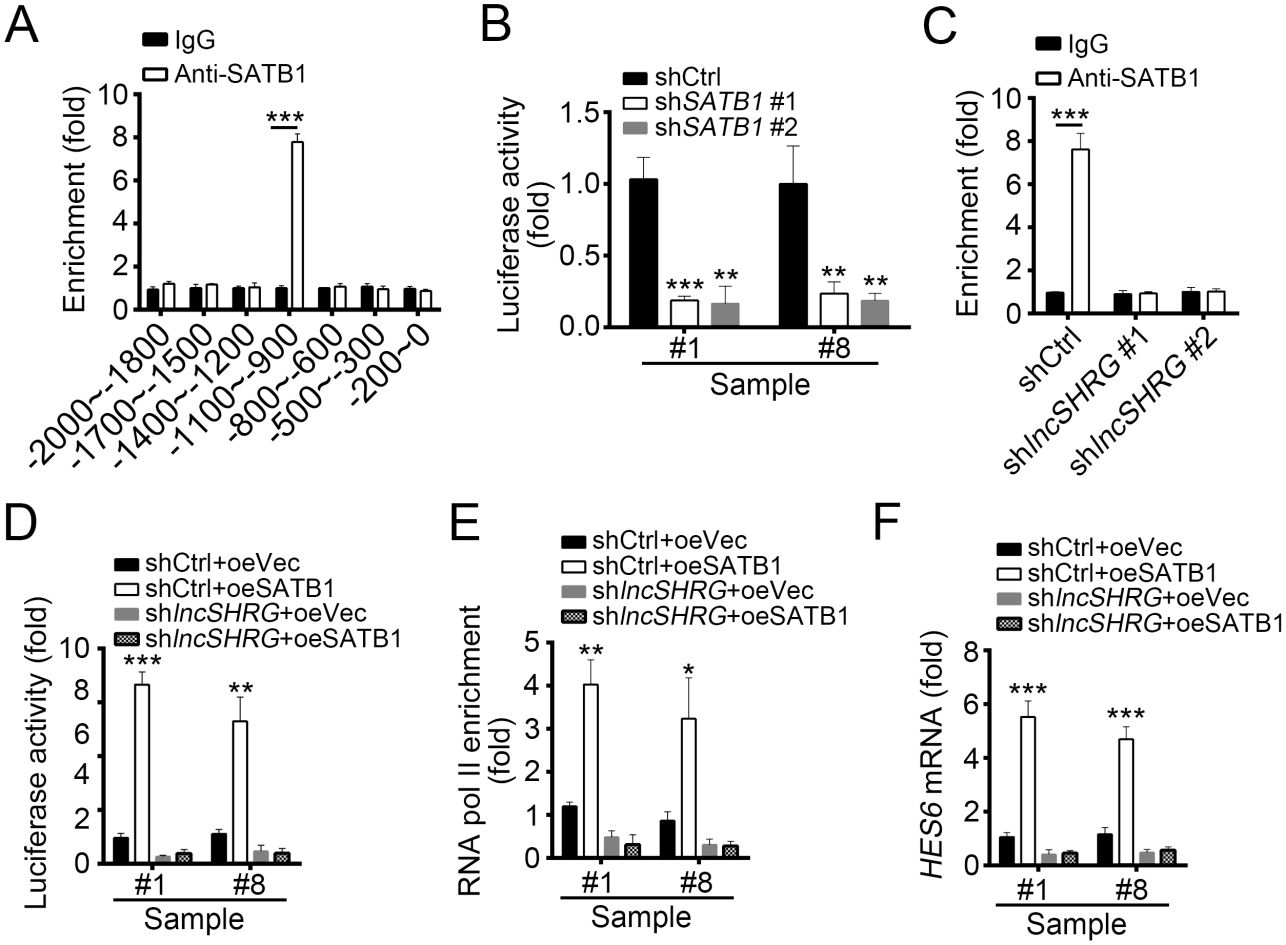
LncSHRG is essential for SATB1 binding to *HES6* promoter **(A)** SATB1 bound to *HES6* promoter (-1100bp∼-900bp) as shown by ChIP assays. χ axis stands for the distance from transcription start site (TSS). **(B)** SATB1 knockdown inhibited *HES6* transcription. Luciferase activity assays were performed with SATB1-silenced sample cells. **(C)** LncSHRG knockdown impaired SATB1 binding to *HES6* promoter (-1100∼-900) as shown by ChIP-qPCR assays. WT or lncSHRG-silenced HCC sample cells were lysed and incubated with anti-SATB1 for ChIP assays. **(D)** LncSHRG was essential for SATB1-regulated *HES6* transcription. Luciferase activity assays were performed with WT or lncSHRG-silenced sample cells. **(E)** SATB1 overexpression promoted enrichment of RNA pol II on *HES6* promoter (-1100∼-900) via an lncSHRG-dependent manner. **(F)** SATB1 promoted *HES6* expression relying on the presence of lncSHRG. WT or lncSHRG-silenced sample cells were transfected with SATB1-overexpressing plasmid or empty plasmid control. Then mRNA levels of HES6 were measured by real time qPCR. *p<0.05, **p<0.01 and ***p<0.001 by two-tailed Student’s t test. All data presented are shown as means ± SD collected from three independent experiments.

We found that lncSHRG interacts with SATB1. To defined the role of SATB1 in lncSHRG-regulated *HES6* expression, we detected whether SATB1 binds to *HES6* promoter by ChIP assays and found that SATB1 was enriched on the same region of *HES6* promoter as lncSHRG (Figure 5A), indicating that SATB1/lncSHRG complex probably regulates *HES6* expression. To further confirm it, we cloned SATB1/ lncSHRG binding region of *HES6* promoter into pGL3 luciferase reporter plasmid and performed luciferase assays. Interestingly, SATB1 knockdown really impaired *HES6* promoter activation (Figure 5B). However, ChIP assays with lncSHRG-depleted sample cell lysates showed that no SATB1 enrichment on *HES6* promoter was observed, proving that lncSHRG was indispensible for the interaction of SATB1 and *HES6* promoter (Figure 5C).

To further demonstrate the role of lncSHRG in SATB1-mediated *HES6* expression, we overexpressed SATB1 in lncSHRG-depleted cells, and then analyzed the activation of *HES6* promoter, chromatin accessibility and *HES6* mRNA levels. We found that SATB1 overexpression promoted *HES6* promoter activation in WT cells but not lncSHRG-silenced cells by luciferase activity reporter assays (Figure 5D). ChIP assays also showed that overexpressing SATB1 enhanced the enrichment of RNA pol II on *HES6* promoter, which indicated a state of transcriptional activation (Figure 5E). However, lncSHRG depletion reversed it (Figure 5E). Similarly, SATB1 overexpression greatly increased the mRNA level of *HES6* in WT cells, but not lncSHRG-silenced cells (Figure 5F). Then we analyzed the correlation of expression between SATB1 and HES6 in HCC sample tissues by RT-qPCR and found that their expressions were positively correlated (Supplementary Figure 1C). Collectively, lncSHRG was essential for SATB1 binding to *HES6* promoter, and SATB1 promoted *HES6* transcription initiation via a lncSHRG-dependent manner.

### HES6 promotes proliferation and is positively correlated with poor prognosis

**Figure 6:**
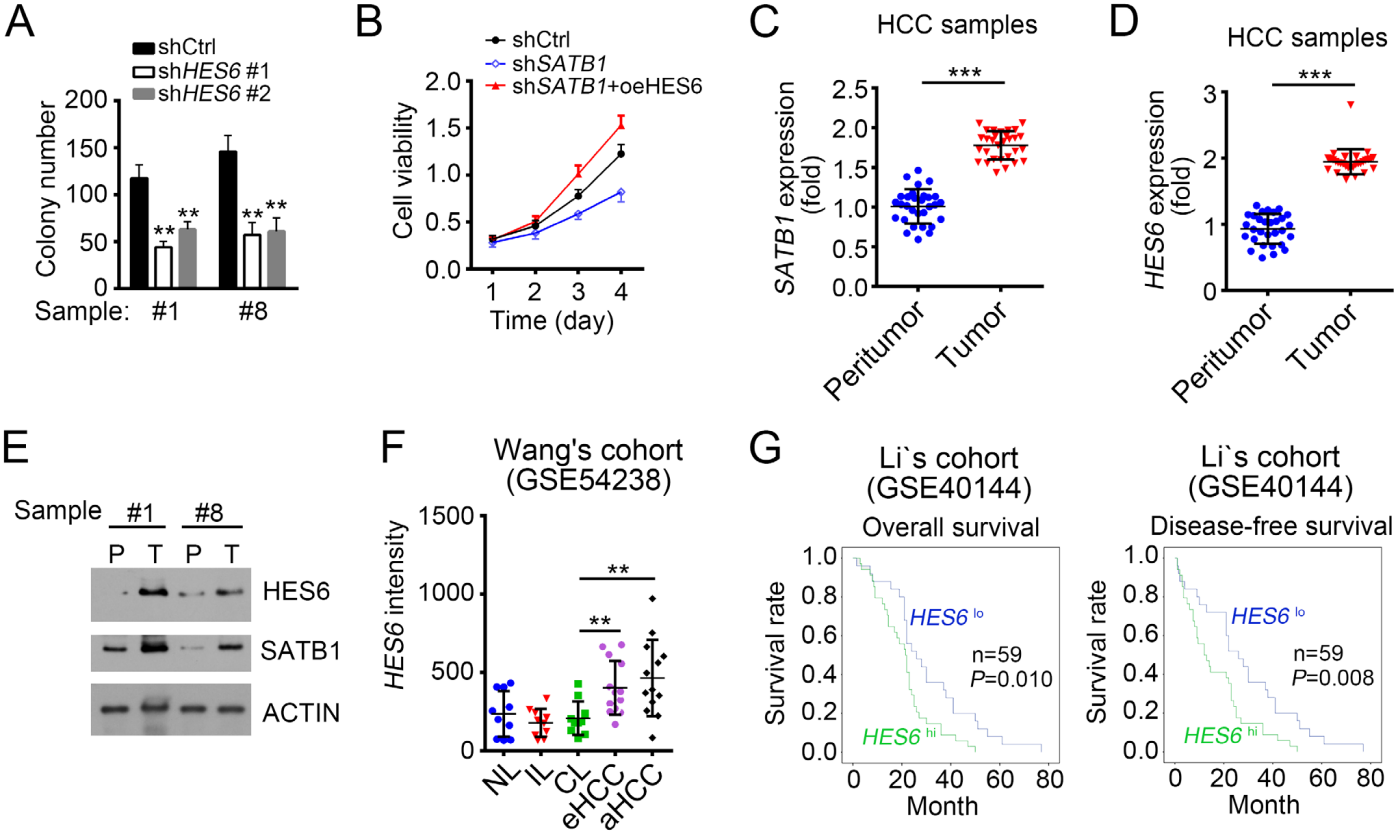
HES6 promotes proliferation and is positively correlated with poor prognosis **(A)** HES6 knockdown inhibited colony formation. HES6 was knocked down for colony formation assays. **(B)** HES6 overexpression promoted cell proliferation. WT or SATB1-silenced sample cells were transfected with HES6-overexpressing plasmid or control. Then MTT assays were conducted. **(C** and **D)** *SATB1* and *HES6* were expressed higher in tumor than peritumor as measured by RT-qPCR. **(E)** *SATB1* and *HES6* expression levels were checked by WB in HCC samples. **(F)** High expression of *HES6* in serious HCC samples as analyzed according to the microarray data in Wang’s cohort (GSE54238). **(G)** *HES6* expression is positively correlated with HCC poor prognosis. Kaplan–Meier survival analyses were performed according to the microarray data in Li’s cohort (GSE40144). *p<0.05, **p<0.01 and ***p<0.001 by two-tailed Student’s t test. All data presented are shown as means ± SD collected from three independent experiments.

To further define the physiological role of SATB1 and HES6 in the regulation of HCC cells, we conducted colony formation assays and MTT assays. We found that HES6 knockdown seriously attenuated the colony formation ability (Figure 6A). Besides, MTT assays showed that SATB1 knockdown inhibited the proliferation of HCC cells while overexpressing HES6 in SATB1-depeted cells enhanced its proliferation capacity obviously (Figure 6B), which indicated that HES6 functions in the downstream of SATB1. To further verify the role of SATB1 and HES6, we examined their expression levels in 30 pairs of samples. We found that SATB1 and HES6 expressed higher in tumor tissues than in peritumor cells (Figure 6C and 6D). We then confirmed it by WB (Figure 6E). We then performed *In vivo* xenograft experiments to confirm the role of HES6. We found that HES6 knockdown Hep3B cells produced smaller tumors than control cells (Supplementary Figure 1D). To further explore the clinical significance of HES6 expression in HCC tumorigenesis and progression, we analyzed the data set of Wang’s cohort (GSE54238), and found higher expression levels of HES6 in eHCC (early HCC) and especially in aHCC (advanced HCC) (Figure 6F). To investigate the relationship of HES6 expression with HCC prognosis, we divided HCC samples into two groups based on HES6 expression levels (mean expression level chosen for cut-off value). Then Kaplan–Meier survival analysis was performed and results indicated that HES6^hi^ patients with HCC had a poor prognosis and lower survival rate, and vice versa (Figure 6G). In summary, lncSHRG recruited SATB1 to *HES6* promoter and then SATB1 drove *HES6* expression, which was essential for HCC cell proliferation.

## DISCUSSION

HCC, one of the most common cancers, has extremely low 5-year survival rate of about 18% [20, 21]. Therefore, large amounts of efforts have been made to investigate the molecular mechanisms of regulating hepatocarcinogenesis and develop methods for HCC diagnosis and treatment. However, what we achieved is very limited and the underlying molecular mechanism of hepatocarcinogenesis remains elusive. Thus, there is an urgent need to reveal the molecular mechanisms underlying HCC progression. In this study, we show that lncSHRG is highly expressed in HCC tissues, especially in advanced HCC samples. LncSHRG knockdown remarkably impairs the proliferation and migration abilities of HCC cells, and leads to cell apoptosis. Moreover, lncSHRG depletion inhibits tumor propagation in mice. In mechanism, lncSHRG recruits SATB1 to *HES6* promoter and LncSHRG (nt 200∼400) was essential for the interaction with SATB1. lncSHRG and SATB1 corporately initiate the expression of *HES6*. Then highly expressed HES6 promotes HCC progression. In a word, the lncSHRG/SATB1/HES6 axis regulates hepatocarcinogenesis.

lncRNAs are longer than 200 nucleotides and have no protein coding potential. They are implicated in a variety of important biological functions such as development and immunomodulation [22–24]. Accumulating evidences show that lncRNAs may play an essential role in physiologic and pathologic processes including tumor progression [25–27], such as HCC [28]. More and more reports show that lncRNA dysregulation is associated with tumor development, metastasis and prognosis [29]. Several lncRNAs have been demonstrated to be involved in HCC. For example, The long noncoding RNA SchLAH is reported to suppresses metastasis of HCC [30]. LncRNA HULC enhances hepatocarcinogenesis by modulating YB-1 phosphorylation [31]. However, the relationship between lncRNAs and HCC is still largely unknown. Large amounts of lncRNAs involved in HCC are not identified. In this study, we screen out an uncharacterized lncRNA called lncSHRG that promotes the proliferation, migration and survival of HCC cells by regulating *HES6* expression. We found that lncSHRG (nt200∼400) was essential for its interaction with SATB and function in HCC. Depletion of nt200∼400 in lncSHRG abrogated its effect on cell proliferation. This region (nt200∼400) in lncSHRG may be important for the tertiary structure, which is pivot to interact with SATB1.

SATB1 has been identified as a chromatin organizer and transcription regulator [32]. SATB1 is reported to modulate large sets of gene expression in a variety of tissues [33]. For example, Satb1 controls HSC self-renewal and lymphoid lineage commitment by upregulating *Myc* expression in mice [34, 35]. In addition, dysregulation of SATB1 expression also results in oncogenesis. SATB1 promotes tumor metastasis in oral squamous cell carcinoma [36]. SATB1 is correlated with breast cancer [37]. In HCC, SATB1 is also reported to promote tumor metastasis and prevents apoptosis [38, 39]. However, the mechanism by which SATB1 functions in HCC need to be explored thoroughly. Here we show that SATB1 binds to *HES6* promoter in HCC tissues and initiates its transcription in the presence of lncSHRG.

HES6, a member of the Hairy/Enhancer-of-split family and a target of Notch signaling, functions as a cofactor and associates with other transcription factors to regulate gene transcription [40]. HES6 has been shown to participate in tumorigenesis. Previous study shows that HES6 enhances prostate cancer aggressiveness in a Notch signalling independent way [15]. Besides, HES6 also promotes the motility of alveolar rhabdomyosarcoma cells [41]. Nevertheless, the role of HES6 in HCC has not been defined. Here we find that HES6 knockdown inhibits HCC cell proliferation seriously. And its expression is regulated by lncSHRG and SATB1. When the Notch signaling was inhibited in Hep3B cells by addition of N-[N-(3,5-difluorophenacetyl)-L-alanyl]-S-phenylglycine t-butyl ester (DAPT), the expression levels of lncSHRG and SATB1 were not changed (Supplementary Figure 1E). Therefore, lncSHRG/SATB1-regulated HES6 expression may be Notch signaling independent. Moreover, HES6 higher expression in HCC patients means lower survival rate and poor prognosis.

Summarily, our study shows that lncSHRG is able to promote cell proliferation and tumor propagation through SATB1/HES6 signaling in HCC. However, whether lncSHRG was highly expressed in other cancer types needs to be further explored. And whether the lncSHRG/SATB1/HES6 axis works in other cancers remains to be elaborated.

## MATERIALS AND METHODS

### Patient samples

30 pairs of peritumor and tumor tissues with HCC used in this study were obtained from Beijing Tongren Hospital. The disease characteristics of all samples used were listed in Supplementary Table 1. Written consents approving the use of their tissues for this research were obtained from all patients. The study protocol was approved by Capital Medical University.

### Cell lines and cell culture

SMMC-7721, BEL-7405 (7405), Huh7, Hep3B human liver cancer cell lines and HL-7702 (7702) normal liver cell line were purchased from Shanghai Cancer Institute (China). The cells were grown in Dulbecco's modified Eagle's medium (DMEM) supplemented with 10% fetal bovine serum (Thermo Fisher, Waltham, MA, USA) and maintained at 37°C in a humidified incubator containing 5% CO2.

### ShRNA-mediated interference

shRNAs against lncSHRG, SATB1 and HES6 were designed using siRNA designer (http://sirna.wi.mit.edu/home.php) and purchased from Sangon Biotech. The effective sequences of shRNAs were as follows: lncSHRG #1: 5'-GACCTGCAGAGATGAATGT-3', #2 5'-CATCCTGCAAGGATATTGT-3'; SATB1: 5'-GCCTCAAACAAGCATTATA-3'; HES6: 5'-GGCGCATTGAGATGCAAAT-3'. shRNAs against lncSHRG, SATB1, HES6 and scramble control were cloned into pSICOR-GFP plasmid. For lentivirus production, 293T cells were transfected with pSICOR-GFP, VSVG, pMDL gp RRE and RSV-REV vectors for 2 days. Then the medium was collected and PEG5000 was added for 2 days at 4°C, and then the virus was collected by centrifugation and used to infect HCC or Hep3B cells. The knockdown cells were isolated and established through FACS by gating on GFP. The knockdown efficiencies of lncSHRG, SATB1 and HES6 were validated by qPCR or Western blot. Hep3B cells or sample cells transfected with pSICOR-GFP-scramble were chosen for control.

### Antibodies

Anti-Satb1 (H-70) and anti-HES6 (sc-133196) were purchased from Santa Cruz Biotechnology. Anti-RNA pol II (4H8) was purchased from Active Motif. Anti-His (6AT18) were purchased from Sigma-Aldrich.

### Apoptosis analysis

Apoptosis analysis was conducted to analyze the apoptotic cells through Annexin V-FITC/PI apoptosis detection kit (eBiosciences). HCC cells were seeded in 6-well plate and transfected with sh*lncSHRG* or shCtrl. 48 h later, cells were collected and stained with Annexin V-FITC/PI according to the manufacturer’s instruction, followed by FACS. Data were analyzed using the FlowJo 7.6.1 software.

### *In vivo* xenograft experiments

Six-week-old male BALB/c nude mice were purchased from HFK Biosciences and maintained under pathogen-free conditions with approval by the Institutional Committee of Capital Medical University. For tumor propagation analysis, 2×10^6^ HCC sample cells, lncSHRG–silenced Hep3B cells or control cells were subcutaneously injected into BALB/c nude mice. Tumor volume was calculated by the formula *V*=π*ab*^*2*^/6 (a: tumour length, b: tumour width) at indicative time points. Tumor weight was measured on week 5 post injection. Animal experiments were performed in accordance with relevant guidelines and regulations of the Institutional Animal Care and Use Committees at Capital Medical University, and protocols were approved by the Institutional Animal Care and Use Committees at Capital Medical University.

### MTT, colony formation and migration assays

In MTT assays, 1×10^3^ cells per well were seeded into 96-well plates. Cell proliferation was measured once every one day. MTT (20 μl, 5 mg/ml) (Sigma, USA) was added into each well at the indicated time points and incubated for 4 h at 37°C. Then 150 μl DMSO was added to solubilize the crystals. To determine cell viability, the absorbance (540 nm) was measured for each well.

For colony formation assays, 2×10^3^ cells were seeded into a 6-well plate and incubated for 12 days at 37°C. And then the cells were fixed in 90% ethanol and stained with crystal violet solution. The formed colonies were counted.

For cell Migration assays, the transwell filter chambers (Costar, Corning, NY) were used according to the manufacturers’ instructions. Briefly, 2×10^5^ cells were resuspended in serum-free medium, and added into the top chamber. Medium with 10% FBS was added to the lower chamber. After incubation for 12 h, cells on the lower surface were stained, photographed, and counted by a microscope in six random fields for each group.

### Northern blot

Total RNA was extracted from HCC samples with TRIzol. 10 μg RNA from each sample was subjected to formaldehyde-denaturing agarose electrophoresis, followed by transferring to positively charged nitrocellulose membrane with 20×SSC buffer (3.0 M NaCl and 0.3 M sodium citrate, pH 7.0). Membrane was UV cross-linked and incubated with hybrid buffer for 2 h prehybridization, followed by incubation with biotin-labeled RNA probes (lncSHRG: nt42∼250). Biotin signals were detected with HRP-conjugated streptavidin according to the manufacturer’s instruction (Thermo Scientific).

### Real-time quantitative PCR (RT-qPCR)

Total RNAs were extracted with TRIzol according to the manufacturer’s protocol. cDNA was synthesized with the M-MLV reverse transcriptase (Promega, Madison, USA). Then mRNA transcripts were analyzed with ABI 7300 qPCR system using specific primer pairs. Relative expressions were calculated and normalized to endogenous *Actb*. Primers used were listed in Supplementary Table 2.

### Chromatin immunoprecipitation (ChIP) assay

Cells were cross-linked with 1% formaldehyde at 37°C for 10 min. Then cells were washed twice with PBS, lysed with SDS lysis buffer (1% SDS, 10 mM EDTA, 50 mM Tris, pH 8.1) and sonicated into 300–500 bp DNA fragments. Lysates were pre-cleared with Protein A Agarose/Salmon Sperm DNA (50% Slurry) and then incubated with 4 μg antibody overnight at 4°C. Then Protein A Agarose/Salmon Sperm DNA (50% Slurry) beads were added for another 4 h. After washed, DNA was eluted from beads and purified. DNA fragments were analyzed by q-PCR with ABI 7300 qPCR system using specific primer pairs. Primers used for *HES6* promoter analysis were listed in Supplementary Table 3.

### EMSA assay

EMSA experiments were conducted according to the manufacturer’s protocol with a Light Shift Chemiluminescent EMSA Kit (Thermo Scientific). Briefly, His-SATB1 was incubated with or without unlabeled probe for competitive reaction and anti-His antibody for super shift at RT for 20 min in a reaction buffer. Then, Biotin-labeled probe (lncSHRG: nt200-400) was added into the reaction system and incubated for 20 min at RT. Samples were carried out in 4% polyacrylamide gel in 0.5 x TBE buffer. After transferred on a nylon membrane (Amersham Biosciences), the labeled DNA was cross-linked by UV, probed with streptavidin-HRP conjugate and then incubated with the detection substrate.

### RNA pulldown and mass spectrometry

For RNA pulldown assays, Biotin-labeled lncSHRG and anti-sense control of lncSHRG were obtained by T7 transcription *in vitro* using Biotin RNA labeling Mix (Roche) and added into cell lysates containing His-SATB1. After incubation overnight at 4°C, Streptavidin-magnetic C1 beads was added and incubated for another 4 h at 4°C. Then biotin-enriched components were separated with SDS–PAGE and examined by WB. For lncSHRG domain mapping, truncated lncSHRGs were cloned into pCDNA3 plasmid. Then truncated lncSHRG DNA fragments were amplified by PCR and acted as templates for T7 transcription *in vitro* with Biotin RNA labeling Mix (Roche). The pulldown assays were perform as described above. For mass spectrometry, the biotin-enriched components were separated with SDS–PAGE and subjected to silver staining. Differential bands enriched by lncSHRG were collected for mass spectrometry (LTQ Orbitrap XL).

### RNA immunoprecipitation (RIP) assay

Cells were treated with 1% formaldehyde and then lysed with RNase-free RIPA buffer (50 mM Tris-HCl, pH 7.4, 150 mM NaCl, 0.5% sodium desoxycholate, 0.1% SDS, 5 mM EDTA, 2 mM PMSF, 20 mg/ml aprotinin, 20 mg/ml leupeptin, 10 mg/ml pepstatin A, 150 mM benzamidine and 1% Nonidet P-40) supplemented with protease-inhibitor cocktail and RNase inhibitor (Roche). Samples were sonicated on ice three times and centrifuged at 12,000g for 10 min. Supernatants were pre-cleared with Protein A/G beads and incubated with antibodies overnight at 4 °C. Protein A/G beads were then added and incubated for 4 h. Total RNA was eluted and extracted. LncSHRG enrichment was checked by qPCR with ABI 7300 qPCR system using specific primer pairs.

### Chromatin isolation by RNA purification

CHIRP-qPCR assays were described previously [42]. In brief, antisense DNA probes against lncSHRG were designed by Biosearch Probe Designer (#1: 5′-tgcaactgccaaagagtctt-3′; #2: 5′-catcttgacacgaggtcatt-3′; #3: 5′-aagcaacttaatgctaggct-3′). Probes were labeled with biotin and purchased from Invitrogen. Beads:biotin-probes:RNA:chromatin were captured by magnets (Invitrogen). Finally, Beads are resolved for DNA with DNA elution buffer (50 mM NaHCO3, 1% SDS, 200 mM NaCl).

### Luciferase reporter gene assays

Luciferase reporter gene assay was performed using the Dual-Luciferase Reporter Assay System (Promega) according to the manufacturer’s instructions. Cells were transferred into 24-well plates at 3×10^4^ cells per well. The indicated regions of *HES6* promoter (-1100∼-900) were cloned into pGL3 luciferase reporter plasmid. 1 ng pRL-TK was co-transfected into cells as loading control. 24 hours later, cells were crashed using lysis buffer and detected for luciferase activity according to manufacturer’s instructions.

### *In situ* hybridization (ISH)

ISH was conducted as previously described protocol [43]. In brief, HCC and peri-tumour samples were fixed with formalin and embedded with paraffin, and sections were obtained. Then the samples were incubated in graded alcohols and incubated in 3% hydrogen peroxide (H2O2) for 30 min. Biotin-conjugated probes and streptavidin-HRP conjugate were used for ISH. Then the samples were co-stained with haematoxylin, followed by dehydration in graded alcohols and xylene. Biotin-conjugated probes were designed using online tools (www.biosearchtech.com) and synthesized by Invitrogen.

### Cohort analysis

Online-available data set analysis has been described before [43]. In brief, the gene expression profiles were analyzed using Excel, SPSS or Graphpad prism 6 software. Three online available cohorts used were as follows. Wang’s cohort (GSE54238) shows microarray data of lncRNAs and genes, containing 10 normal liver samples, 10 inflammatory liver samples, 10 cirrhosis liver samples, 13 early HCC samples and 13 advanced HCC samples. Li’s cohort (GSE40144) has microarray data with disease-free survival and overall survival information of 59 hepatocellular carcinoma. Wang’s cohort (GSE14520) has gene microarray data with survival information containing two normal liver samples, 228 peritumour and 239 liver cancer samples.

### Statistical analysis

For box and whisker plots, figures were produced with GraphPad Prism 6 software. Box indicates interquartile range; whiskers indicate 5–95 percentiles; horizontal line within box denotes median value. *P*-values were calculated by two-tailed Student’s t-test with GraphPad Prism 6 software, and *P*<0.05 was considered significant. For histograms, data were shown as means ± SD. Two-tailed Student’s t-test was used for statistical analysis with GraphPad Prism 6 software. *P<0.05; **P<0.01; ***P<0.001. *P*<0.05 was considered significant.

## SUPPLEMENTARY MATERIALS FIGURE AND TABLES



## References

[1] Bruix J, Gores GJ, Mazzaferro V (2014). Hepatocellular carcinoma: clinical frontiers and perspectives. Gut.

[2] Wang YY, Qi LN, Zhong JH, Qin HG, Ye JZ, Lu SD, Ma L, Xiang BD, Li LQ, You XM (2017). High expression of AKR1B10 predicts low risk of early tumor recurrence in patients with hepatitis B virus-related hepatocellular carcinoma. Sci Rep.

[3] Correnti M, Raggi C (2017). Stem-like plasticity and heterogeneity of circulating tumor cells: current status and prospect challenges in liver cancer. Oncotarget.

[4] Oikawa T (2016). Cancer stem cells and their cellular origins in primary liver and biliary tract cancers. Hepatology.

[5] Morise Z, Kawabe N, Tomishige H, Nagata H, Kawase J, Arakawa S, Yoshida R, Isetani M (2014). Recent advances in the surgical treatment of hepatocellular carcinoma. World J Gastroenterol.

[6] Wang KC, Yang YW, Liu B, Sanyal A, Corces-Zimmerman R, Chen Y, Lajoie BR, Protacio A, Flynn RA, Gupta RA, Wysocka J, Lei M, Dekker J (2011). A long noncoding RNA maintains active chromatin to coordinate homeotic gene expression. Nature.

[7] Zhu P, Wang Y, Huang G, Ye B, Liu B, Wu J, Du Y, He L, Fan Z (2016). lnc-β-Catm elicits EZH2-dependent β-catenin stabilization and sustains liver CSC self-renewal. Nat Struct Mol Biol.

[8] Luo S, Lu JY, Liu L, Yin Y, Chen C, Han X, Wu B, Xu R, Liu W, Yan P, Shao W, Lu Z, Li H (2016). Divergent lncRNAs regulate gene expression and lineage differentiation in pluripotent cells. Cell Stem Cell.

[9] Atianand MK, Caffrey DR, Fitzgerald KA (2017). Immunobiology of long noncoding RNAs. Annu Rev Immunol.

[10] Qi F, Liu X, Wu H, Yu X, Wei C, Huang X, Ji G, Nie F, Wang K (2017). Long noncoding AGAP2-AS1 is activated by SP1 and promotes cell proliferation and invasion in gastric cancer. J Hematol Oncol.

[11] Deng J, Yang M, Jiang R, An N, Wang X, Liu B (2017). Long non-coding RNA HOTAIR regulates the proliferation, self-renewal capacity, tumor formation and migration of the cancer stem-like cell (CSC) subpopulation enriched from breast cancer cells. PLoS One.

[12] Xia M, Yao L, Zhang Q, Wang F, Mei H, Guo X, Huang W (2017). Long noncoding RNA HOTAIR promotes metastasis of renal cell carcinoma by up-regulating histone H3K27 demethylase JMJD3. Oncotarget.

[13] Seta Y, Seta C, Barlow LA (2003). Notch-associated gene expression in embryonic and adult taste papillae and taste buds suggests a role in taste cell lineage decisions. J Comp Neurol.

[14] Haapa-Paananen S, Kiviluoto S, Waltari M, Puputti M, Mpindi JP, Kohonen P, Tynninen O, Haapasalo H, Joensuu H, Perälä M, Kallioniemi O (2012). HES6 gene is selectively overexpressed in glioma and represents an important transcriptional regulator of glioma proliferation. Oncogene.

[15] Carvalho FL, Marchionni L, Gupta A, Kummangal BA, Schaeffer EM, Ross AE, Berman DM (2015). HES6 promotes prostate cancer aggressiveness independently of Notch signalling. J Cell Mol Med.

[16] Roessler S, Long EL, Budhu A, Chen Y, Zhao X, Ji J, Walker R, Jia HL, Ye QH, Qin LX, Tang ZY, He P, Hunter KW (2012). Integrative genomic identification of genes on 8p associated with hepatocellular carcinoma progression and patient survival. Gastroenterology.

[17] Roessler S, Jia HL, Budhu A, Forgues M, Ye QH, Lee JS, Thorgeirsson SS, Sun Z, Tang ZY, Qin LX, Wang XW (2010). A unique metastasis gene signature enables prediction of tumor relapse in early-stage hepatocellular carcinoma patients. Cancer Res.

[18] Yuan SX, Wang J, Yang F, Tao QF, Zhang J, Wang LL, Yang Y, Liu H, Wang ZG, Xu QG, Fan J, Liu L, Sun SH, Zhou WP (2016). Long noncoding RNA DANCR increases stemness features of hepatocellular carcinoma by derepression of CTNNB1. Hepatology.

[19] Fatica A, Bozzoni I (2014). Long non-coding RNAs: new players in cell differentiation and development. Nat Rev Genet.

[20] Allemani C, Weir HK, Carreira H, Harewood R, Spika D, Wang XS, Bannon F, Ahn JV, Johnson CJ, Bonaventure A, Marcos-Gragera R, Stiller C, Azevedo e Silva G (2015). Global surveillance of cancer survival 1995-2009: analysis of individual data for 25,676,887 patients from 279 population-based registries in 67 countries (CONCORD-2). Lancet.

[21] Altekruse SF, McGlynn KA, Dickie LA, Kleiner DE (2012). Hepatocellular carcinoma confirmation, treatment, and survival in surveillance, epidemiology, and end results registries, 1992-2008. Hepatology.

[22] Vallot C, Patrat C, Collier AJ, Huret C, Casanova M, Liyakat Ali TM, Tosolini M, Frydman N, Heard E, Rugg-Gunn PJ, Rougeulle C (2017). XACT noncoding RNA competes with XIST in the control of X chromosome activity during human early development. Cell Stem Cell.

[23] Kotzin JJ, Spencer SP, McCright SJ, Kumar DBU, Collet MA, Mowel WK, Elliott EN, Uyar A, Makiya MA, Dunagin MC, Harman CC, Virtue AT, Zhu S (2016). The long non-coding RNA Morrbid regulates Bim and short-lived myeloid cell lifespan. Nature.

[24] Turner M, Galloway A, Vigorito E (2014). Noncoding RNA and its associated proteins as regulatory elements of the immune system. Nat Immunol.

[25] Chen R, Wang G, Zheng Y, Hua Y, Cai Z (2017). Long non-coding RNAs in osteosarcoma. Oncotarget.

[26] Gutschner T, Diederichs S (2012). The hallmarks of cancer: a long non-coding RNA point of view. RNA Biol.

[27] Pan Y, Li C, Chen J, Zhang K, Chu X, Wang R, Chen L (2016). The emerging roles of long noncoding RNA ROR (lincRNA-ROR) and its possible mechanisms in human cancers. Cell Physiol Biochem.

[28] Yang X, Xie X, Xiao YF, Xie R, Hu CJ, Tang B, Li BS, Yang SM (2015). The emergence of long non-coding RNAs in the tumorigenesis of hepatocellular carcinoma. Cancer Lett.

[29] Qiu MT, Hu JW, Yin R, Xu L (2013). Long noncoding RNA: an emerging paradigm of cancer research. Tumour Biol.

[30] Ge Z, Cheng Z, Yang X, Huo X, Wang N, Wang H, Wang C, Gu D, Zhao F, Yao M, Fan J, Qin W (2017). The long noncoding RNA SchLAH suppresses metastasis of hepatocellular carcinoma through interacting with FUS. Cancer Sci.

[31] Li D, Liu X, Zhou J, Hu J, Zhang D, Liu J, Qiao Y, Zhan Q (2017). Long noncoding RNA HULC modulates the phosphorylation of YB-1 through serving as a scaffold of extracellular signal-regulated kinase and YB-1 to enhance hepatocarcinogenesis. Hepatology.

[32] Yasui D, Miyano M, Cai S, Varga-Weisz P, Kohwi-Shigematsu T (2002). SATB1 targets chromatin remodelling to regulate genes over long distances. Nature.

[33] Cai S, Han HJ, Kohwi-Shigematsu T (2003). Tissue-specific nuclear architecture and gene expression regulated by SATB1. Nat Genet.

[34] Will B, Vogler TO, Bartholdy B, Garrett-Bakelman F, Mayer J, Barreyro L, Pandolfi A, Todorova TI, Okoye-Okafor UC, Stanley RF, Bhagat TD, Verma A, Figueroa ME (2013). Satb1 regulates the self-renewal of hematopoietic stem cells by promoting quiescence and repressing differentiation commitment. Nat Immunol.

[35] Satoh Y, Yokota T, Sudo T, Kondo M, Lai A, Kincade PW, Kouro T, Iida R, Kokame K, Miyata T, Habuchi Y, Matsui K, Tanaka H (2013). The Satb1 protein directs hematopoietic stem cell differentiation toward lymphoid lineages. Immunity.

[36] Li YC, Bu LL, Mao L, Ma SR, Liu JF, Yu GT, Deng WW, Zhang WF, Sun ZJ (2017). SATB1 promotes tumor metastasis and invasiveness in oral squamous cell carcinoma. Oral Dis.

[37] Pan Z, Jing W, He K, Zhang L, Long X (2016). SATB1 is correlated with progression and metastasis of breast cancers: a meta-analysis. Cell Physiol Biochem.

[38] Liu J, Han P, Li M, Yan W, Liu J, Liu J, He J, Tu W, Xia Y, Zhou Z, Gong J, Liu M, Ding Q, Tian D (2015). The histidine-rich calcium binding protein (HRC) promotes tumor metastasis in hepatocellular carcinoma and is upregulated by SATB1. Oncotarget.

[39] Kuo TC, Chao CC (2010). Hepatitis B virus X protein prevents apoptosis of hepatocellular carcinoma cells by upregulating SATB1 and HURP expression. Biochem Pharmacol.

[40] Pissarra L, Henrique D, Duarte A (2000). Expression of hes6, a new member of the Hairy/Enhancer-of-split family, in mouse development. Mech Dev.

[41] Wickramasinghe CM, Domaschenz R, Amagase Y, Williamson D, Missiaglia E, Shipley J, Murai K, Jones PH (2013). HES6 enhances the motility of alveolar rhabdomyosarcoma cells. Exp Cell Res.

[42] Chu C, Qu K, Zhong FL, Artandi SE, Chang HY (2011). Genomic maps of long noncoding RNA occupancy reveal principles of RNA-chromatin interactions. Mol Cell.

[43] Chen ZZ, Huang L, Wu YH, Zhai WJ, Zhu PP, Gao YF (2016). LncSox4 promotes the self-renewal of liver tumour-initiating cells through Stat3-mediated Sox4 expression. Nat Commun.

