# Supplementary material for: LncSHRG promotes hepatocellular carcinoma progression by activating HES6
Source: Oncotarget. 2017 Aug 3;8(41):70630–41. doi: 10.18632/oncotarget.19906 (PMC5642582; doi:10.18632/oncotarget.19906)
Supplement: Supplementary file 1 [file oncotarget-08-70630-s001.pdf]

# LncSHRG promotes hepatocellular carcinoma progression by activating *HES6*

## SUPPLEMENTARY MATERIALS

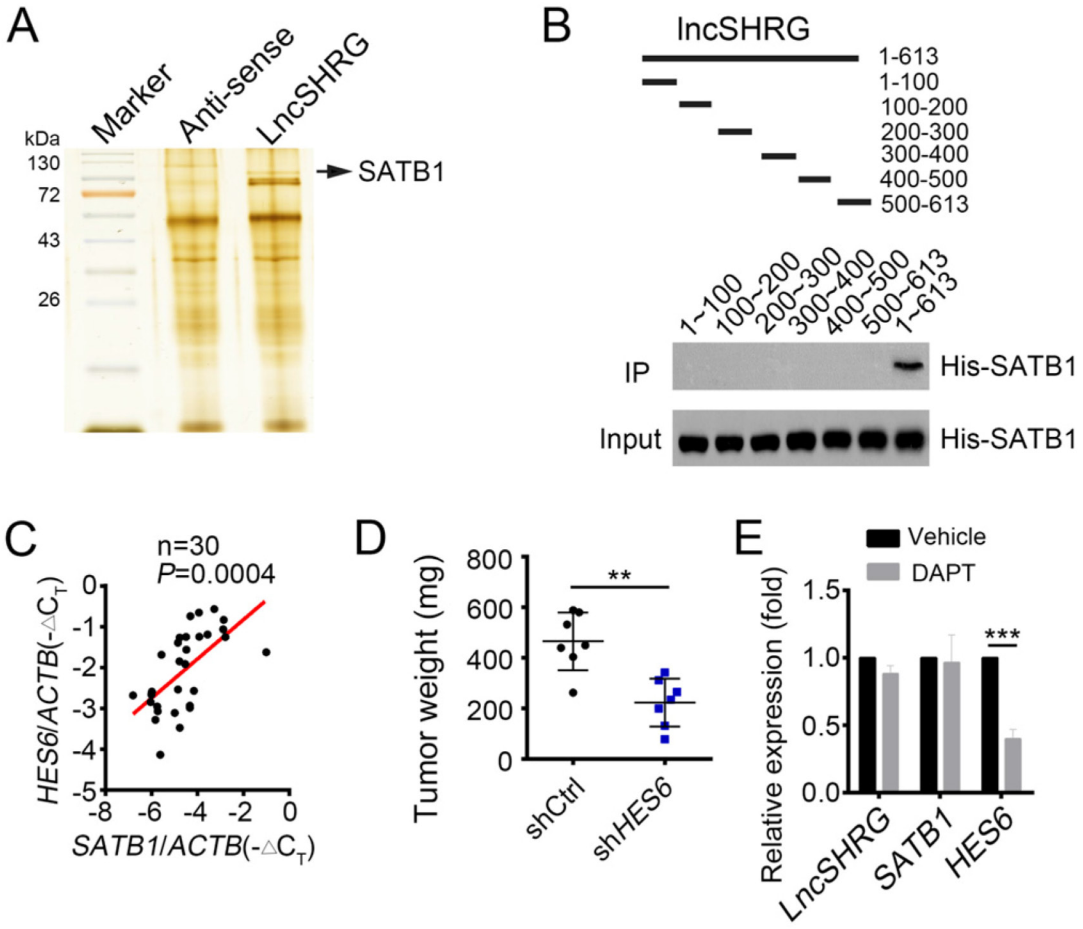


**Supplementary Figure 1: LncSHRG interacts with SATB1. (A)** Biotin-RNA pulldowns were performed using biotin-labeled lncSHRG or anti-sense control. Eluted fractions were resolved by SDS-PAGE, followed by silver staining and mass spectrometry. SATB1 was identified as a potential interactive protein of lncSHRG. **(B)** Analysis of the interactive region in lncSHRG with SATB1 by domain mapping and RNA pulldown assays. LncSHRG truncates were obtained by T7 transcription *in vitro* and biotin labeled. **(C)** *SATB1* expression was positively correlated with that of *HES6* in HCC samples. **(D)** Analysis of mRNA levels of lncSHRG, SATB1 and HES6. HCC sample cells were cultured with in the presence of N-[N-(3,5-difluorophenacetyl)-L-alanyl]-S-phenylglycine t-butyl ester (DAPT) (1 μM) or not. DAPT was an inhibitor of Notch signaling. **(E)** $2 \times 10^6$ HES6-depleted or control Hep3B cells were injected into nude mice. The weights of tumors were measured on week 5 after injection. **p<0.01 by two-tailed Student's t test. All data presented are shown as means ± SD collected from three independent experiments.

**Supplementary Table 1: Information of clinical patients**

| Sample no. | Age | Gender | Diagnosis | Tumor size(cm) | Stage |
|---|---|---|---|---|---|
| 1# | 40 | Male | HCC | 20*12.5*8.5 | advanced |
| 2# | 60 | Female | HCC | 2.5*2.5*1.5 | middle-ad |
| 3# | 54 | Male | HCC | 8.4*8.2*7.6 | middle-ad |
| 4# | 62 | Male | HCC | 2.6*2.3*1.8 | early-mid |
| 5# | 37 | Male | HCC | 10*8*8 | early-mid |
| 6# | 56 | Male | HCC | 5*4.5*4 | advanced |
| 7# | 53 | Male | HCC | 9*9*6.5 | middle-ad |
| 8# | 54 | Male | HCC | 6*5.8*5.5 | middle-ad |
| 9# | 31 | Female | HCC | 9.5*8.5*5.8 | middle-ad |
| 10# | 41 | Male | HCC | 2.5*1.5*2 | early |
| 11# | 36 | Female | HCC | 2.2*1.2*1 | early |
| 12# | 54 | Male | HCC | 13*11*9 | middle-ad |
| 13# | 68 | Female | HCC | 2.5*2.4*1.8 | early |
| 14# | 64 | Female | HCC | 1.9*1.5*1 | early |
| 15# | 51 | Male | HCC | 2.2*1.7*1.6 | early-mid |
| 16# | 50 | Male | HCC | 6.5*5.5*4 | early-mid |
| 17# | 43 | Male | HCC | 3*2.5*2 | early-mid |
| 18# | 53 | Male | HCC | 3*3*2 | early-mid |
| 19# | 49 | Male | HCC | 8.5*5.5*4.1 | middle-ad |
| 20# | 71 | Male | HCC | 4*3*3 | early-mid |
| 21# | 76 | Male | HCC | 11*8*7.5 | middle-ad |
| 22# | 60 | Male | HCC | 3*3*2.5 | early-mid |
| 23# | 60 | Male | HCC | 1.2*1*0.9 | early |
| 24# | 61 | Male | HCC | 7*7*5.5 | middle-ad |
| 25# | 42 | Male | HCC | 4.3*4.5*4 | early-mid |
| 26# | 49 | Male | HCC | 4*2.5*3 | early |
| 27# | 61 | Male | HCC | 10*7*6 | middle-ad |
| 28# | 57 | Male | HCC | 4*3*3 | advanced |
| 29# | 61 | Female | HCC | 3*2.5*2 | advanced |
| 30# | 49 | Male | HCC | 2.5*2.2*2.1 | early |

**Supplementary Table 2: Real time PCR primers used in this study**

| Genes | Forward | Reverse |
|---|---|---|
| *lncSHRG* | 5’-TCAACAATTAAGACTCTTTGGCAGT-3’ | 5’-CTGCCATCTTGACACGAGGT-3’ |
| *Actb* | 5’-TCCATCATGAAGTGTGACGT-3’ | 5’-GAGCAATGATCTTGATCTTCAT-3’ |
| *HIF1a* | 5’-GAACGTCGAAAAGAAAAGTCTCG-3’ | 5’-CCTTATCAAGATGCGAACTCACA-3’ |
| *VEGF* | 5’-ATCACGAAGTGGTGAAGTTC-3’ | 5’-TGCTGTAGGAAGCTCATCTC-3’ |
| *TWIST1* | 5’-GTCCGCAGTCTTACGAGGAG-3’ | 5’-GCTTGAGGGTCTGAATCTTGCT-3’ |
| *MYC* | 5’-GGCTCCTGGCAAAAGGTCA-3’ | 5’-CTGCGTAGTTGTGCTGATGT-3’ |
| *CCND2* | 5’-TTTGCCATGTACCCACCGTC-3 | 5’-AGGGCATCACAAGTGAGCG-3’ |
| *TCF1* | 5’-CTGGCTTCTACTCCCTGACCT-3’ | 5’-ACCAGAACCTAGCATCAAGGA-3’ |
| *HES6* | 5’-AGCAGGAGCCTGACTCAGTT-3’ | 5’-AGCTCCTGAACCATCTGCTC-3’ |
| *HEY1* | 5’-GTTCGGCTCTAGGTTCCATGT-3’ | 5’-CGTCGGCGCTTCTCAATTATTC-3’ |
| *NRARP* | 5’-TCAACGTGAACTCGTTCGGG-3’ | 5’-ACTTCGCCTTGGTGATGAGAT-3’ |
| *GLI1* | 5’-TGGATATGATGGTTGGCAAGTG-3’ | 5’-ACAGACTCAGGCTCAGGCTTCT-3’ |
| *PTCH1* | 5’-CCACAGAAGCGCTCCTACA-3’ | 5’-CTGTAATTTCGCCCCTTCC-3’ |
| *GLI3* | 5’-GAAGTGCTCCACTCGAACAGA-3’ | 5’-GTGGCTGCATAGTGATTGCG-3’ |
| *Satb1* | 5’-TGATTTGTGACGCACCCTCA-3’ | 5’-AGCAAGGCAGAAACATCCCC-3’ |

**Supplementary Table 3: Primers used for *HES6* promoter analysis in this study**

| Region | Forward | Reverse |
|---|---|---|
| -2000~-1800 | 5'-TTAATGTACACAAATCAGTAGC-3' | 5'-CATCGTAGAGGTCTTTTGATTCC-3' |
| -1700~-1500 | 5'-ACAAGAGCGAAACTCCCTCTG-3' | 5'-GGGATTGCATTGAATTTGTAGATT3' |
| -1400~-1200 | 5'-AAGGCCATAGTTACCAAAACAG-3' | 5'-ATGTGTTGAAAAGGGTGTCCTT-3' |
| -1100~-900 | 5'-CTAGAAGATAACACTGGATAA-3' | 5'-TTACTCTACTGACTGTTCCCT-3' |
| -800~-600 | 5'-TAATCCCTAACCCCCAACCT-3' | 5'-AAGGTTGGTGTTAGGGGTTAA-3' |
| -500~-300 | 5'-AATGCTGCATTTGTCTTCCGAGGC-3' | 5'-AGTGTGGAAAACAGGAAACCA-3' |
| -200~0 | 5'-AGGAGAACGCTGCTCTGCCTTCAC-3 | 5'-CGAGGGCGGAGCAGTGTTCT-3' |
